# Enhancement of modern defense capabilities by convergence of cognitive neuroscience and artificial intelligence

**DOI:** 10.3389/fpsyg.2026.1821242

**Published:** 2026-06-09

**Authors:** Kresimir Cosic, Anđela Kuljanac, Sara Palermo

**Affiliations:** 1 Faculty of Electrical Engineering and Computing, University of Zagreb, Zagreb, Croatia; 2 School of Medicine, University of Zagreb, Zagreb, Croatia; 3 Department of Psychology, University of Turin, Turin, Italy; 4 Neuroscience Institute of Turin (NIT), Turin, Italy; 5 AI@UniTo, University of Turin's Scientific Platform on Artificial Intelligence, University of Turin, Turin, Italy

**Keywords:** artificial intelligence, cognitive neuroscience, mental readiness, modern defense, stress resilience

## Abstract

In the context of evolving geopolitical instability, hybrid threats, and the increasing relevance of the cognitive domain in modern conflict, there is a growing need to better understand the role of human cognition in defense and security affairs. Advances in cognitive neuroscience provide insights into the neural and psychological mechanisms underlying decision-making, stress resilience, attention, and metacognition, while artificial intelligence (AI) offers new capabilities for data integration, predictive modeling, and decision support. This article proposes a multi-level conceptual framework for integrating cognitive neuroscience and AI as a hierarchical cognitive support system that enhances, rather than replaces, human decision-making under conditions of uncertainty, time pressure, and cognitive load. The integration of soft neurocognitive science and AI capabilities with kinetic hard power may strengthen deterrence, resilience, and long-term defense sustainability. The proposed interdisciplinary framework can facilitate the development of more adaptive, resilient, and human-centered approaches to defense and security in complex and volatile environments.

## 1 Introduction

In the face of accelerating geopolitical instability, kinetic and hybrid threats, and the return of high-intensity kinetic warfare, the limits of traditional defense paradigms and doctrines have become strategically visible. The contemporary wars have exposed critical gaps in modern defense architecture regarding its military capability, as well as cognitive preparedness, strategic decision-making and overall societal resilience. At the same time, the rapid integration of Artificial Intelligence (AI) into defense and security systems has opened new opportunities to enhance military effectiveness, situational awareness, and strategic anticipation. However, the mere adoption of AI technologies does not automatically translate into strategic advantage if the human cognitive dimension is not explicitly addressed and integrated within operational and governance frameworks.

A new paradigm related to the integration of cognitive neuroscience and artificial intelligence to strengthen modern kinetic military power through a deeper understanding of human cognition in augmentation of strategic decision-making capacity of civil and military leadership is emerging with an unprecedented level of complexity. This shift marks a transition from artificial intelligence as a technological enabler to cognitive domain as an increasingly relevant operational dimension, where AI and neuroscience jointly support the protection, enhancement, and sustainment of human cognition across military, political, and societal domains.

In this context, AI should be understood not as a substitute for human judgment, but as a cognitive amplifier that supports human decision-making under conditions of uncertainty, stress, time pressure, and information overload (Palermo, 2020a, 2020b), consistent with human-centered AI and human-AI teaming approaches (Shneiderman, 2020; Gonzalez et al., 2026; Kargarnovin et al., 2026). In an era of hybrid threats, disinformation, and psychological manipulation, modern defense can no longer depend on kinetic power. Its strategic effectiveness may increasingly depend on human cognitive readiness, emotional regulation, and decision-making capacities. Traditional defense models based on kinetic power require integration with AI enhanced cognitive capabilities to cope with the surge of cognitive disinformation, emotional and cognitive manipulation, and contamination of informational space as strategic risks. On a societal level, adversaries now exploit psychological instruments to target the foundations of our democracy and civil society, alter our perception of democracy, trust in institutions, polarize public discourse, and manipulate democratic processes. In terms of manipulation, it is important to mention the sensitization effect, which is an increase in responsiveness to a stimulus when it is repeated. In this context, past traumatic events can sensitize individuals, leading to greater distress when consuming related media (Thompson et al., 2019), which can then be used as a form of manipulation. These vulnerabilities are consistent with broader literature showing that misinformation uptake and resistance to correction are shaped by cognitive, affective, and identity-related processes (Ecker et al., 2022; Lewandowsky and van der Linden, 2021). Cognitive neuroscience provides a scientific framework to understand these vulnerabilities of human cognition, while AI enables scalable, real-time analysis of information dynamics and cognitive stressors across complex environments. However, despite its potential, this domain remains under-recognized in modern defense policy and public discourse in order to prevent future psychological devastation of civil and military population.

AI systems could analyze how adversaries’ disinformation spreads across modern media and social networks (Cinelli et al., 2022; Marigliano et al., 2024; Pacheco et al., 2021; Sharma et al., 2021), allowing our defense and security policymakers to respond faster and more effectively. Neither cognitive neuroscience nor AI can replace sound democratic political judgment and human reasoning, but their institutional integration can enhance situational awareness, mitigate cognitive biases, and preserve decision integrity under pressure and uncertainties. This convergence of AI and cognitive neuroscience represents a conceptual evolution from technology-centered defense architectures toward cognitively informed, human-centered defense systems. Integration of cognitive neuroscience and AI into the modern defense and security affairs promises to create more predictive, adaptive, resilient, and effective strategies to effectively cope with the complexities of modern threats. This interdisciplinary approach can enhance our operational capabilities, improve operational effectiveness, enhance mental resilience, mental well-being of the military, improve overall mission outcomes, and ultimately ensure greater security and stability. It can also promote a deeper understanding of the human factors, like understanding of human cognitive limitations, such as cognitive overload, fatigue, and stress, that may reduce defense capabilities and dynamics.

Therefore, to meet these 21st-century threats, integration of cognitive neuroscience and AI into modern warfare capabilities should be based on fostering collaboration between defense agencies, academia, and industry. Such integration should include AI-driven cognitive monitoring, decision support, and cognitive training systems as core components of modern defense architectures. This suggests that these capabilities may be interpreted as a collective strategic asset, distributed across political, scientific, military, and technological institutions, since defense success will depend not only on traditional military capabilities, but also on an integrated cognitive capacity enhanced by AI. This perspective reflects a shift from artificial intelligence as a technological tool to cognitive capabilities as a system-level objective. This perspective also suggests a possible framework through which modern warfare may conceptualize and manage future defense challenges, by unlocking the full potential of human cognition in tandem with AI and traditional kinetic military power.

Cognitive neuroscience should assist in a more comprehensive understanding of human behavior in military strategic contexts, providing innovative insights into perception, emotion, cognitive stress, fatigue, mental health, stress resilience, attention, working memory, cognitive overload, situational awareness, decision-making capacities and operational readiness which are increasingly recognized as key operational defense capabilities (Ćosić et al., 2022; Palermo, 2020a, 2020b, 2020c). Neuroscience also reveals how military leaders manage stress, cognitive bias, uncertainty, complex warfare scenarios, and how repeated exposure to propaganda shapes human belief systems (Hebb’s rule, “Fire Together-Wire Together”), while AI’s real-time analytical capacity and predictive simulations, based on vast amounts of data can identify patterns and predict potential military threats and respond more effectively in crisis situations on tactical, operational, and strategic levels.

This interdisciplinary approach, combining cognitive neuroscience and AI, holds significant potential for enhancing modern defense strategic decision-making capacities, and for creating more sophisticated predictive models capable of addressing the complexity of future warfare operations. It suggests that the future of modern defense may not be determined solely by kinetic firepower, but increasingly by human factor (Fletcher and Wind, 2013; Grier, 2012), grounded in the integration of cognitive neuroscience and AI (Taddeo and Floridi, 2018).

**Figure 1:**
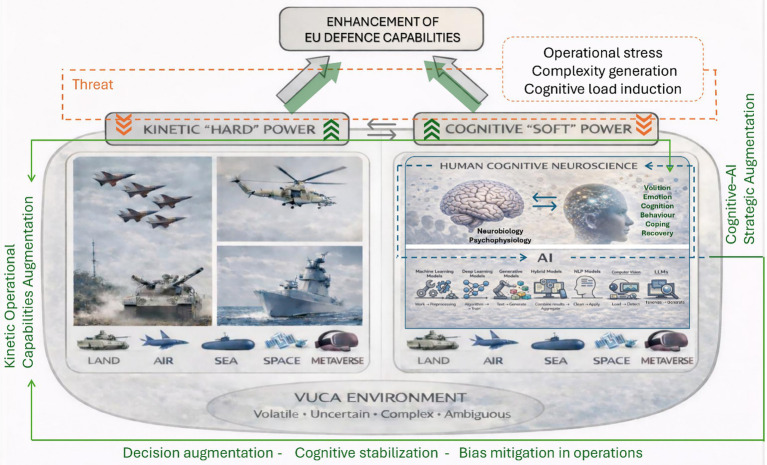
Integrated neuro-AI defense architecture within a VUCA strategic environment.

To improve conceptual clarity, the proposed framework is structured across multiple levels of analysis, spanning neural mechanisms, individual cognitive processes, collective dynamics, and strategic governance, which are explicitly integrated throughout the manuscript. This paper aims to provide a conceptual multi-level framework for the integration of cognitive neuroscience and artificial intelligence into modern defense and security affairs, outlining how neurocognitive insights, AI-based analytics, and adaptive cognitive training can jointly contribute to enhancing cognitive readiness and strategic adaptability (Figure 1).

The diagram illustrates the interaction between kinetic operational capabilities and cognitive–AI strategic augmentation across modern warfare domains (land, air, sea, space, cyber, and metaverse). Within a volatile, uncertain, complex, and ambiguous (VUCA) context, operational pressures generate cognitive load and stress, while neurocognitive mechanisms supported by AI-driven monitoring and decision support enhance cognitive readiness and decision integrity under stress.

In “Mind Wars: Brain Research and National Defense” (Moreno, 2006), author discussed potential of neuroscience in military applications, including modifying soldiers’ memory, cognition, emotions, behavior, and mental capacities, while in the 1990s, the U.S. National Academy of Sciences began mapping neural and anatomical brain connections, exploring molecular and genetic mechanisms of underlying brain function (Institute of Medicine (U.S.) Committee on a National Neural Circuitry Database, 1991; McCreight, 2024). Today, facts and findings are related to numerous papers on: neurocognitive factors of new drone pilots (Ramallo-Luna et al., 2025), human factors and AI in unmanned aerial vehicle systems (Alharasees and Kale, 2025), applications of machine learning in assessing cognitive load of uncrewed aerial system operators (Li et al., 2025), etc. Additionally, forward looking scenarios might lead to overall integration of AI, ML, DL, and LLM tools and means into integrated warfare operations along entire hierarchical chain of C2 and C4ISR system of systems and developments of corresponding ontologies (Bebber, 2025; Schubert et al., 2018; Trabucco and Larsen, 2025).

## 2 Cognitive warfare and the human domain

In contemporary security environments, warfare increasingly extends beyond physical and cyber domains into the cognitive domain, where human perception, attention, emotions, beliefs, and decision-making processes become primary targets. This evolution has been formally recognized within NATO as cognitive warfare, defined as the strategic exploitation of the human domain to influence, disrupt, degrade, or manipulate individual and collective cognition in order to achieve political and military objectives (Cole and Le Guyader, 2020; Claverie and Du Cluzel, 2022).

According to NATO Allied Command Transformation, cognitive warfare represents a paradigm shift in which the human mind becomes both the battlespace and the weapon, encompassing psychological, informational, social, and neurocognitive dimensions. This framing has been articulated within NATO strategic foresight initiatives emphasizing the cognitive domain as an operational battlespace (Cole and Le Guyader, 2020; Claverie and Du Cluzel, 2022). Unlike traditional psychological operations, cognitive warfare is continuous, multi-layered, and operates across military, political, and civilian spheres, targeting not only adversaries’ forces but also its leadership, institutions, and populations. Its ultimate objective is not immediate physical destruction, but the systematic erosion of trust, situational awareness, decision integrity, and societal cohesion.

Research conducted over the past decade at the intersection of military and civilian domains has demonstrated that cognitive warfare is not a transient phenomenon, but a structural feature of modern conflict (Claverie and Du Cluzel, 2022). Recent analyses emphasize that cognitive warfare constitutes a continuous form of strategic competition aimed at shaping how populations think rather than merely what they think (Pastor, 2025). Long-term interdisciplinary investigations highlight how stress, uncertainty, information overload, and emotional contagion can amplify cognitive vulnerabilities at both individual and collective levels. The weaponisation of neuroscientific knowledge, combined with distributed computing infrastructures, has been identified as a defining element of this emerging domain (Pastor, 2025).

These vulnerabilities are further exacerbated by the speed and scale of digital information environments, where repeated exposure to emotionally charged narratives can progressively shape beliefs, perceptions, and behavioral responses (Pomerantsev, 2019). From a cognitive and social psychology perspective, collective behavior plays a critical role in the effectiveness of cognitive warfare. Classical and contemporary theories of crowd psychology show that individuals embedded in groups are particularly susceptible to emotional contagion, perceptual distortion, and heuristic-driven judgments. Under conditions of fear, uncertainty, and perceived threat, collective cognition may shift toward polarization, simplification of complex realities, and reduced critical thinking, making populations more vulnerable to manipulation (Palermo, 2020c). These mechanisms are increasingly exploited through coordinated information campaigns that leverage repetition, emotional salience, and identity-based narratives to influence public perception and decision-making at scale.

In this context, cognitive warfare directly challenges traditional defense models that prioritize kinetic capabilities and technological superiority while underestimating the human cognitive factor (Pastor, 2025). The effectiveness of military operations, strategic leadership, and societal resilience depends not only on physical assets, but also on the capacity of individuals and groups to maintain cognitive stability, emotional regulation, and coherent decision-making under sustained pressure. Consequently, protecting and strengthening the human domain emerges as a strategic necessity in modern warfare.

Understanding cognitive warfare therefore requires a systematic integration of cognitive neuroscience, psychology, and artificial intelligence. Neuroscience provides insights into the mechanisms underlying perception, emotion, stress responses, and belief formation, while AI enables large-scale monitoring, analysis, and modeling of cognitive and behavioral dynamics in complex information environments. Together, these disciplines offer the foundations for developing defensive strategies aimed at detecting cognitive attacks, mitigating their effects, and enhancing cognitive resilience across military and civilian domains (Claverie and Du Cluzel, 2022; Pastor, 2025). Importantly, these processes operate across interconnected levels of analysis, from neural and individual cognition to collective social dynamics and broader strategic environments, requiring a multi-level interpretative framework.

## 3 Neuro-cognitive foundations of modern defense capabilities

High operational capabilities and performance during contemporary conflict environments characterized by cognitive warfare, uncertainty and unpredictability, including high-intensity, hybrid and information-driven operations, are decisive factors in modern warfare (Matthews et al., 2019; National Research Council, 2015; Smith et al., 2019; Vernon et al., 2007). These operational capabilities and effectiveness depend on the integrity and stability of the human cognitive domain, including situational awareness, decision-making, perception, attention, resilience, working memory and adaptability under sustained cognitive and emotional pressure. From this perspective, neurocognitive mechanisms should not be interpreted in isolation, but as foundational processes that scale from individual performance to collective behavior and ultimately to strategic decision-making.

In the context of cognitive warfare, these cognitive functions are not only determinants of performance, but also primary targets of adversarial action aimed at degrading human judgment and decision integrity. This implies that cognitive degradation in warfare contexts is not accidental but may represent a strategically engineered outcome of adversarial influence operations targeting human neural and psychological vulnerabilities. The convergence of cognitive neuroscience and AI may therefore play a significant role in future defense, improving military effectiveness by explaining how distress, combat fatigue, and cognitive overload affect soldiers’ performance, revealing how soldiers process stressful combat information, form situational awareness, shape beliefs, and make right decisions or fall into cognitive biases (Brown and Morey, 2012; Taddeo and Floridi, 2018).

State of the art supporting tools, like virtual assistant (VA) or adaptive human machine interface (AHMI) can enable soldiers’ dynamic adaptation to mission-critical stressful tasks and enhance their mental readiness by integration and optimization of combat information flow, reducing cognitive overload and emotional exhaustion, improving situational awareness, and increasing operational effectiveness and unit cohesion. Within cognitively contested environments, such systems may also serve a defensive role by stabilizing human cognition against manipulation, overload, and stress-induced degradation.

The proposed multi-level conceptual framework presented in this article addresses the integration of cognitive neuroscience and AI as a hierarchical cognitive support system from the lowest neuronal level related to brain dynamics, neuronal plasticity, dendritic and synaptic interactions, neural wiring diagrams, firing patterns with C2 and C4ISR systems that may assist in the construction of a common operational picture, threat analysis, course-of-action comparison, and decision support under time pressure. It discusses relationship between human decision-making capabilities under conditions of uncertainty, time pressure, and cognitive load with strategic policy-level decision-making processes. At this strategic level, integration of cognitive neuroscience and AI may support scenario analysis, information fusion, policy assessment, and crisis decision-making during stressful warfare operations while still requiring meaningful human judgment, legal accountability, uncertainty communication, and meaningful human control.

### 3.1 Key cognitive functions

**Figure 2:**
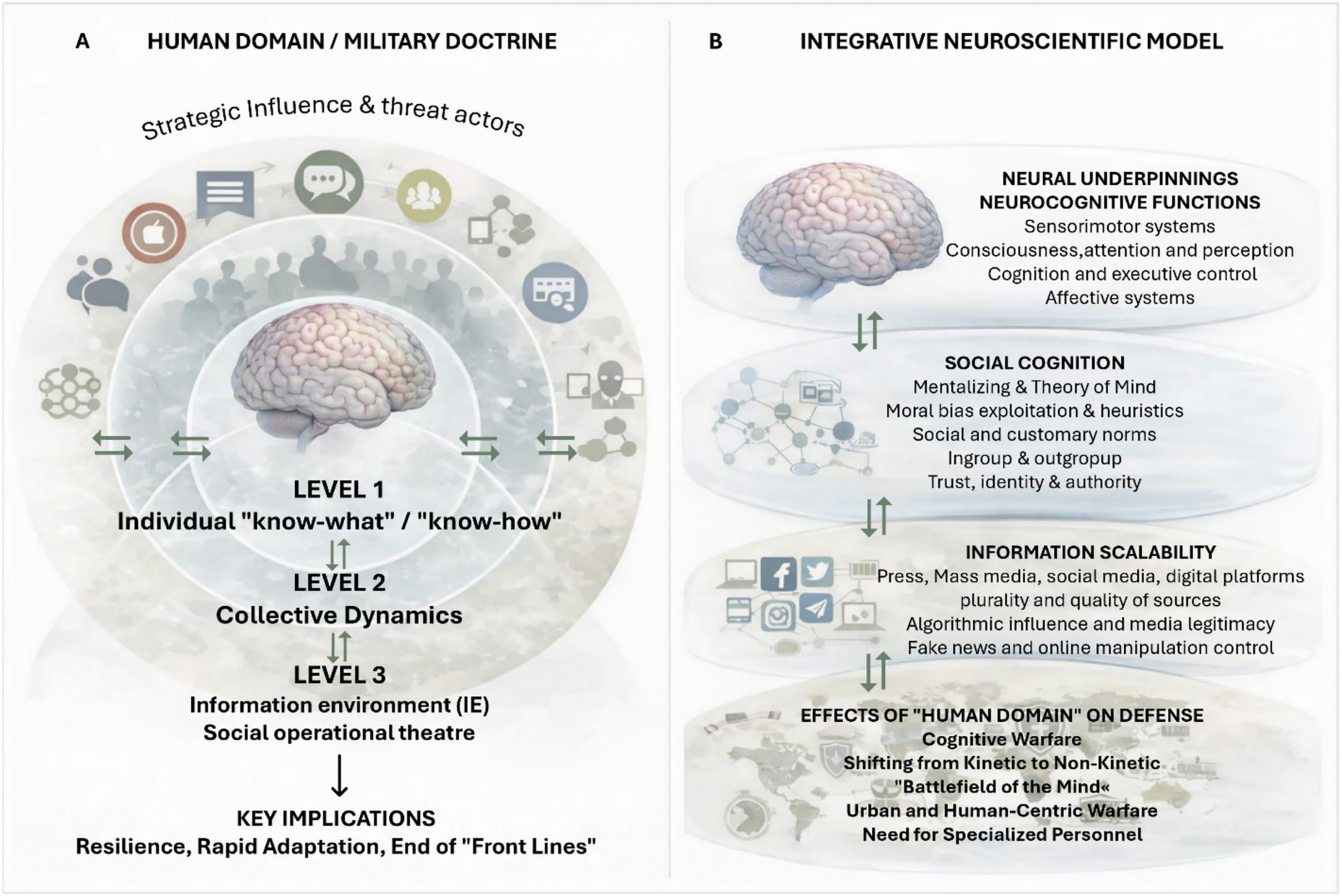
Human domain and integrative neuroscientific model of cognitive warfare and defense capability.

High cognitive operational capabilities depend on several key cognitive conditions or processes that all together sustain higher-order cognitive processes vital for defense effectiveness and higher defense capabilities (Figure 2).

*Panel A (left): Human domain/military doctrine multi-level structure*. This panel conceptualizes the human domain as a three-level architecture operating within contemporary information and social operational theatres.

- Level 1—Individual “know-what”/“know-how.”
  This level refers to the individual cognitive foundation of military capability. *Know-what* includes doctrinal knowledge, theoretical frameworks, and factual information relevant to military operations. *Know-how* encompasses practical skills, technical proficiency, procedural competence, and experiential wisdom acquired through training and operational exposure.
- Level 2—Collective dynamics.
  This intermediate level captures the social and relational dimension of military functioning, including unit cohesion, social bonding, informal status hierarchies, peer influence, mentorship, organizational culture, and norms. It also reflects social learning processes and group behavior under high-stress conditions, which shape operational performance beyond individual competence.
- Level 3—Information environment and social operational theatre.
  At the systemic level, the information environment (IE) influences both individual and collective cognition. This includes media ecosystems, algorithmic amplification, information pollution, psychological operations, strategic communications, and cross-cultural interactions (Ćosić et al., 2012b). These dynamics affect military “know-what” (situational awareness and doctrinal interpretation), “know-how” (tactical expertise and decision-cycle integrity), and even the evolution of military doctrine itself. At the geopolitical scale, this level interacts with great power competition, gray-zone strategies, societal trust, demographic influence, and attribution challenges.

Together, these three levels illustrate how cognitive warfare operates across nested layers of individual cognition, collective social structure, and the broader information environment, ultimately shaping military doctrine and strategic influence.

*Panel B (right): Integrative neuroscientific model*. This panel connects neural underpinnings (sensorimotor systems, executive control, affective systems), social cognition processes (mentalizing, norm processing, trust, identity), and information scalability mechanisms to the operational effects of cognitive warfare. It highlights the shift from purely kinetic confrontation toward non-kinetic influence operations within the “battlefield of the mind,” emphasizing resilience, rapid adaptation, and the need for specialized cognitive competencies.

In the framework of cognitive warfare, these cognitive processes constitute the core of human cognitive capabilities that adversaries seek to influence, disrupt, or exploit (Albertella et al., 2023; Ćosić et al., 2025). The authors analyse the ability to perform optimally under pressure across many occupations, including the military, first responders, and police. The following constructs reached consensus across four panels according to following ranking: (1) Attention and perception; (2) Emotion regulation; (3) Working memory; and (4) Cognitive control; This expert consensus is critical to standardize cognitive assessment in the broader field of human performance optimization. Within cognitively contested operational environments, degradation of these functions may be deliberately induced to impair performance and decision-making (Palermo, 2018). Therefore, the most relevant cognitive features for success in operational stressful environment will be shortly analyzed.

#### 3.1.1 Attention and perception

Stress can narrow attention, leading to hyperfocus on immediate threats while impairing the ability to process peripheral information reducing the effectiveness of military operation (Lieberman et al., 2005). In cognitively contested environments, such attentional narrowing may be externally amplified through information overload and emotionally salient stimuli (Palermo, 2019). This can result in overlooking critical details or potential hazards. Focusing on relevant information while filtering out distractions is crucial for strategic thinking and reasoning. This enables decision-makers to concentrate on the most critical factors affecting their tasks or strategy by selective resource allocation under information overload (Posner and Petersen, 1990). Sustaining vigilance, selective attention under mental and physical overload is the ability to selectively allocate resources to relevant stimuli while resisting distractions, particularly under information overload. Perception is the ability to gather and interpret information from the environment. Effective perception allows leaders to recognize patterns, trends, and changes that may impact strategic decisions. Manipulation of perceptual cues and attentional resources represents a core tactic of cognitive warfare aimed at degrading judgment and increasing error likelihood.

#### 3.1.2 Situational awareness

Situational awareness (SA) is the ability to recognize and understand the current state of the environment and predict state changes in the near future (Endsley, 1995). It is an important basis for determining whether the operator’s state is competent for the current complex task. In cognitive warfare, situational awareness represents a primary target, as adversaries seek to distort perception, fragment information, and undermine coherent interpretation of reality.

More generally, situational awareness in the modern highly complex and volatile world requires high attention and non-bias perception in order to maintain reliable and accurate situational awareness in dynamic environments. Accurate and reliable prediction of situational awareness must be grounded on reliable pattern recognition, cognitive clarity, good judgment, and decisions without cognitive biases, mental overload, and emotional reactivity. It needs real-time integration of multisensory information into coherent defense operational pictures, enabling operation anticipation and adequate rapid response. Disruption of this integration process constitutes a key mechanism through which cognitive attacks impair operational effectiveness.

#### 3.1.3 Working memory

Retaining and recalling past experiences, lessons learned, and historical data is essential for informed decision-making. Memory aids in recognizing successful strategies and avoiding past mistakes. Memory systems are related to episodic memory for contextual recall and understanding, semantic for conceptual frameworks, and working memory for learning, adaptation and real-time data manipulation (Baddeley, 2012). However, high operational performance also depends on some other important high-level cognitive capabilities, like intelligence, critical thinking, creativity, problem-solving, emotion regulation, motivation, learning, and adaptability. In cognitively contested environments, working memory overload may fragment reasoning and impair adaptive flexibility. Neuroplasticity underlies skill acquisition and flexibility (Draganski and May, 2008) allowing the nervous system to reorganize connections in response to training, experience, and novel threats.

#### 3.1.4 Decision-making and reasoning

Decision-making is a “high-level” cognitive process based on more basic cognitive processes such as perception, memory, and attention, through which a preferred option or a course of action is chosen from among a set of alternatives based on certain criteria. Literature shows that stress, affect, and uncertainty systematically shape judgment quality, risk processing, and reliance on heuristics in high-stakes environments (Lerner et al., 2015; Reale et al., 2023). The process of making choices among alternatives involves weighing pros and cons, assessing risks, and considering potential outcomes when military personnel are faced with large amounts of data and information that must be processed in a timely manner, which significantly increases their cognitive load.

Cognitive warfare directly targets decision-making processes by exploiting cognitive biases, emotional stress, and information asymmetries, thereby increasing the probability of suboptimal or maladaptive choices. Therefore, decision-making under the stress and uncertainty when leaders face ambiguous and high-stakes choices, might be strengthened by state-of-the-art research in cognitive neuroscience. This body of work helps clarify how the human brain processes information, evaluates options, and makes decisions, as well as how attention, working memory, and cognitive capacity, vital for mission success, can influence strategic decisions within turbulent and volatile environment. Understanding how the brain processes information can improve decision-making quality in high-stakes environments which relies on cognitive flexibility, strategic foresight, risk calibration, emotional regulation, and metacognitive awareness. Techniques like functional MRI (fMRI) and electrophysiological recordings enable to identify neural correlates of decision-making, revealing how different brain areas contribute to choices in evaluating complex operational scenarios under time pressure.

#### 3.1.5 Metacognition

Metacognition, i.e., thinking about thinking as awareness of one’s own cognitive processes, limits, and biases, enables humility, adaptive learning, and resilient leadership. In cognitive warfare contexts, metacognitive awareness represents a critical defensive capability, enabling individuals and leaders to recognize manipulation, regulate cognitive responses, and maintain decision integrity under sustained pressure.

#### 3.1.6 Cognitive workload and cognitive biases

Research concerning the management of mental workload, attention, and stress is of special interest in many stressful professional jobs. Recognizing conditions in which a human is over-challenged or cannot act lucidly could avoid serious outcomes. Furthermore, knowing in depth a human’s neurophysiological and cognitive–behavioral responses could allow for the optimization of equipment and procedures to minimize risk and increase safety. In cognitive warfare scenarios, excessive cognitive workload may be deliberately induced to overwhelm decision-makers and reduce analytical capacity.

Understanding the cognitive load on policy makers, managers, and soldiers during missions or tasks can help design better operational plans and communication strategies to prevent overload and enhance performance. Assessing mental workload as a multidimensional context-dependent construct is generally done by a combination of self-report, performance, and physiological indicators rather than by any single measure alone (Young et al., 2015; Longo et al., 2022; Hart and Staveland, 1988). As AI systems become more skillful at predicting human behavior, ethical concerns arise regarding privacy, manipulation, and decision autonomy. Understanding the cognitive processes can help ensure that AI applications are developed responsibly and transparently. In the context of cognitive warfare, these ethical risks become more salient (Palermo, 2018, 2019), because predictive and persuasive technologies may be misused to influence beliefs, emotions, and choices.

Virtual assistant based on cognitive neuroscience can aid in strategic planning by modeling potential outcomes based on human cognitive biases and decision-making limitations. Cognitive biases are systematic patterns of deviation from norm or rationality in judgment, and they can significantly influence human decision-making. It might be the tendency to search for, interpret, and remember information that confirms one’s preexisting beliefs or hypotheses while ignoring evidence that contradicts them. It might be the reliance on the first piece of information encountered (the “anchor”) when making decisions. Likewise, it might be the tendency for individuals to overestimate their own abilities, knowledge, or the accuracy of their predictions. In cognitive warfare, such biases may be actively exploited by adversaries to shape perceptions and decisions at individual and collective levels. Understanding these cognitive biases can help individuals and organizations make more informed and rational decisions by recognizing when these cognitive distortions may be influencing their judgment.

### 3.2 Neuroanatomical foundations of modern defense capabilities

The advanced neuroscientific research on cognitive dynamics, key neural functions, brain biochemistry, neuromodulation, and plasticity deserves more attention in the context of cognitive research, as other elements in the brain’s operational environment, like dendrites, nerve cells, axons, and synapses, that govern or influence human thinking, perception, judgment, reasoning, functionality, plasticity, and proprioceptive behavior., Although the precise causal sequences and biochemical mechanisms underlying these processes are still only partially understood, and many unanswered questions still exist. Nevertheless, cognitive operational functions are based on coordinated activity of key distributed neural networks rather than isolated brain regions (Anderson et al., 2021).

Importantly, these neurocognitive systems are not static: they are dynamically modulated by experience, stress, training, and environmental demands, and are therefore amenable to adaptive modulation and targeted cognitive training. The following neuroanatomic networks play critical roles in sustaining operational performance.

#### 3.2.1 Anterior cingulate cortex, amygdala, and hippocampus

The Anterior cingulate cortex (ACC) supports conflict monitoring and error detection, enabling adaptive behavioral adjustments (Botvinick et al., 2019; Miller and Cummings, 2018; Palermo et al., 2018). From a military perspective, the ACC represents a strategically critical neural hub because it mediates the detection of competing demands, operational conflicts, and errors under conditions of uncertainty and stress (Palermo, 2020a), directly supporting rapid behavioral adaptation, performance monitoring, and decision correction in high-stakes operational environments.

Amygdala and hippocampus govern emotional regulation and reactivity, memory consolidation, contextual awareness, spatial navigation, (Eichenbaum, 2017; Pessoa, 2015), regulate threat detection and emotional salience (Pessoa, 2015). They are critical for rapid threat detection, emotional salience attribution, and stress responses, while also interacting with cortical areas to shape situational awareness.

Hippocampus is responsible for memory consolidation, spatial navigation, and contextual awareness, crucial for learning from past operations and projecting future scenarios in operational contexts (Eichenbaum, 2017). Basal ganglia enable procedural learning, habit formation, and reinforcement-driven behaviors (Doya, 2008), i.e., reinforcement-based decision-making, allowing operators to execute complex behaviors automatically under pressure.

These limbic and paralimbic structures, including the anterior cingulate cortex, are central nodes in the neural circuitry targeted during cognitive warfare, as emotional salience, fear conditioning, and stress reactivity can be externally amplified or manipulated. At the same time, their functional connectivity with prefrontal regions can be strengthened or stabilized through targeted training and adaptive regulation strategies.

#### 3.2.2 Precuneus and parietal cortex

Parietal Cortex and Precuneus govern visuospatial coordination, attention distribution (Corbetta and Shulman, 2002), and the integration of sensory information necessary for situational awareness; and Sensory Cortices relay, and prioritize sensory information, maintaining processing efficiency under overload (Sherman, 2016).

Acting as relay hubs, these regions filter and prioritize sensory inputs under conditions of high information load (Sherman, 2016). Furthermore, structures such as the basal ganglia, parietal cortex, and thalamus support procedural learning, visuospatial coordination, and sensory integration (Corbetta and Shulman, 2002; Doya, 2008; Sherman, 2016).

Given their role in attentional allocation and multisensory integration, these regions operate within dynamic large-scale networks, including the salience and executive control networks, which underpin cognitive flexibility and situational readiness (Anderson et al., 2021; Menon, 2015).

#### 3.2.3 Prefrontal cortex

Prefrontal cortex (PFC) plays a central and strategic role in cognitive operational capabilities due to its involvement in various higher-order cognitive processes and functions. It is essential for the complex cognitive processes that underpin effective cognitive operational capabilities, influencing how individuals navigate challenges and make strategic decisions. The PFC is responsible for executive functions, which include planning, organizing, and prioritizing tasks. This enables individuals to set goals and develop strategies to achieve them. It also integrates information from different sources, evaluates options, assesses risks, and weighs potential outcomes, making it crucial for informed decision-making.

It facilitates the identification of problems, generation of possible solutions, and selection of the most appropriate course of action, allowing effective resolution of challenges. The PFC is critical for working memory, which involves temporarily holding and manipulating information. This ability is essential for reasoning, learning, and complex task execution. The PFC regulates attention, allowing individuals to focus on relevant information while filtering out distractions. This is vital in environments with competing stimuli.

It plays a role in managing emotions and controlling impulses (Palermo et al., 2017), which can influence decision processes and social interactions within organizational settings. The PFC aids in understanding social dynamics, including empathy, perspective-taking, and moral reasoning, which are important for collaboration and teamwork. The PFC allows cognitive flexibility, enabling individuals to adapt their thinking and strategies in response to new information or changing circumstances (Miller and Cummings, 2018; Pessoa, 2015).

Crucially, prefrontal executive functions are highly sensitive to stress, fatigue, and cognitive overload, but also exhibit experience-dependent plasticity, making them key targets for AI-supported cognitive training and resilience-building interventions. This strategic relevance is particularly evident in command-and-control contexts, where executive dysfunction under stress may propagate errors across hierarchical decision systems.

### 3.3 Neurobiological and functional networks of modern defense capabilities

#### 3.3.1 Neurobiological mechanisms and neurotransmitter systems

Neurobiological mechanisms and neurotransmitter systems ranging from neurotransmitter regulation, like, dopamine, norepinephrine, serotonin, and acetylcholine to stress physiology related to hypothalamic-pituarity-adrenal axis or HPA axis and neural oscillations—provide the substrates for adaptability and resilience (Buzsáki, 2006; McEwen, 2007; Robbins and Arnsten, 2009). Dopamine modulates motivation and reinforcement learning; norepinephrine regulates arousal and vigilance; serotonin contributes to impulse control and mood stabilization; acetylcholine enhances attention and memory encoding (Robbins and Arnsten, 2009).

These neuromodulatory systems operate in a highly dynamic and state-dependent manner, continuously adjusting neural gain, signal-to-noise ratio, and network efficiency in response to stress, fatigue, and environmental demands. From a military perspective, this dynamic regulation constitutes a critical substrate for both vulnerability to cognitive degradation and potential enhancement through targeted training, recovery protocols, and AI-supported adaptive regulation.

#### 3.3.2 Neuroplasticity and neural oscillations

Synaptic and network-level changes provide the substrate for acquisition of new knowledge and expertise, cognitive adaptability, through synaptic modification and network reorganization sustaining long-term adaptation (Kolb and Whishaw, 2015). Strengthening synapses and network reconfiguration enable acquisition of repetitive skills in response to military training. Brain rhythms in the theta, alpha, and gamma bands coordinate large-scale coordination of attention, memory, and rapid response (Buzsáki, 2006), communication, supporting functions such as memory encoding, attentional gating, and rapid response coordination.

From a mechanistic perspective, these processes are grounded in Hebbian plasticity, whereby repeated co-activation of neural populations strengthens synaptic efficacy (“cells that fire together, wire together”), and in the dynamic regulation of cortical excitability, which reflects the balance between excitatory and inhibitory processes underlying learning, adaptation, and stress responsiveness (Palermo et al., 2025).

Recent evidence suggests that cortical excitability can be conceptualized as a sensitive and malleable biomarker of cognitive resilience, capturing the brain’s capacity to adapt to prolonged cognitive and emotional demands across the lifespan (Palermo et al., 2025). In military populations, this is particularly relevant when considering the entire occupational cycle, from active service and sustained operational stress exposure to post-service transition and veteran status, where long-term cognitive resilience becomes a critical determinant of functional autonomy and mental health (Palermo, 2018, 2020b, 2021a, 2021b).

Accordingly, preserving and appropriately modulating cortical excitability through training, recovery, and adaptive interventions may support resilience not only during active duty, but also in mitigating long-term cognitive vulnerability in veterans, reinforcing the strategic importance of neuroscience-informed cognitive training across the full life-course of military personnel.

#### 3.3.3 Default mode, salience, and executive large-scale networks

Default mode, salience and executive large-scale networks facilitate task switching, self-monitoring (Morese et al., 2018), and environmental scanning (Anderson et al., 2021; Menon, 2015). They also enable dynamic switching between internally directed cognition and external task demands, facilitating cognitive flexibility and readiness, self-monitoring, and environmental scanning (Menon, 2015).

In particular, self-monitoring emerges from the dynamic interaction between the salience network and executive control networks, allowing individuals to continuously evaluate their internal cognitive states, detect performance errors or mismatches between intended and actual behavior, and adjust actions accordingly. This capacity is critical for maintaining goal-directed behavior under uncertainty and stress, as it supports awareness of cognitive limits, error detection, and adaptive control (Morese et al., 2018).

From a military and security perspective, effective self-monitoring is essential for sustaining operational performance in cognitively contested environments, as it enables operators and leaders to recognize cognitive drift, fatigue-related performance degradation, and susceptibility to manipulation, thereby supporting timely corrective actions and resilient decision-making.

## 4 Operational stress, stress resilience, and mental readiness in modern warfare

An increase in operational effectiveness in modern warfare through better stress resilience training and enhancement of mental preparedness and readiness may provide overall mission success. Therefore, it is important to analyze the impact of stress, fatigue, and high cognitive workload in high-pressure environments on situational awareness, attention, perception, multitasking abilities, mental health, strategic decision-making capacity and cognitive performance (Palermo, 2020a, 2020b, 2021a). Stress can reduce cognitive flexibility, making it harder to adapt to changing circumstances or to consider alternative solutions (Palermo, 2020b). This rigidity can limit problem-solving abilities in dynamic environments, and therefore neuro-psycho-physiological prevention strategies for protecting and promoting of mental readiness, mental agility, preparedness and mental health are extremely important. While stress has a strong influence on human cognition, i.e., human decision-making process, it has no influence on potential virtual assistant system as human adviser. Understanding the effects of stress on cognitive performance is crucial in high-stakes environments, such as military operations, emergency response, and high-level decision-making roles. The limitation of the human cognitive system in very complex, turbulent and stressful military environment is restricted due to several interrelated factors, like:

- Human working memory can only hold a limited amount of information at a time (typically 4–7 chunks), while complex multitasking problems often require simultaneous tracking many variables and relationships, which can easily exceed this restricted human capacity, particularly during high stress Human brain processing speed is relatively low, which limits its performance to rapidly evaluate multiple complex scenarios, simulate outcomes mentally or to process and fuse large data streams in real time.
- High levels of stress can slow down cognitive processing speed, making it difficult to respond quickly to rapidly evolving situations (Palermo, 2018, 2019, 2020b). This delay can be crucial in time-compressed military operations (Palermo, 2020a).
- High stress levels can obstruct the retrieval of information from memory networks. Under pressure, individuals may struggle to recall previously learned material or important procedures, which can be detrimental in critical situations.
- Stress can negatively affect interpersonal communication and collaboration, which are vital in high-pressure situations. Misunderstandings and conflicts may arise, further complicating complex situations.
- Humans are prone to cognitive biases (e.g., confirmation bias, availability heuristic) that distort reasoning and problem-solving, especially in unfamiliar or abstract contexts.
- Highly negative arousal, like frustration and anger, might provoke revenge which could lead to unethical actions, preventing right decision or even decision paralysis (van Diggelen et al., 2023).
- Emotional stressors, motivational deficit, and fear of failure can cloud judgment or prevent people from approaching a problem rationally or thoroughly and impede effective communication and teamwork. Stress can impair emotional regulation, leading to heightened anxiety or irritability.
- Limitations of language as a primary tool for thought can make it difficult to express highly complex problems, ambiguities, and vagueness in language, and can obstruct clear reasoning during unexpected stressful situations.
- Acute stress activates the body’s fight-or-flight response, releasing hormones like cortisol and adrenaline. While these can enhance short-term performance, its prolonged exposure can be detrimental to cognitive functions.
- Finally, chronic stress can lead to cognitive fatigue and burnout, diminishing overall cognitive performance over time. This can result in an decreased vigilance and increased likelihood of errors.

Therefore, strategies to manage stress and mitigate its negative impacts on cognitive functioning deserve special attention. From a strategic standpoint, unmanaged stress represents not only an individual vulnerability but a systemic risk for collective decision integrity within defense organizations.

### 4.1 Stress resilience training

Advanced technologies such as virtual and augmented reality, as well as AI and new wearable noninvasive sensors, have pivotal role in the modernization of military training based on realistic immersive training simulations that mirror the complexities of the contemporary battlefield (Popović et al., 2009; Ćosić et al., 2010). This approach aims to contribute to the optimization of military training, enhancement of operational readiness and well-being of armed forces personnel. New wearable noninvasive technologies available today offer a tremendous opportunity to acquire human’s multimodal neuro-psycho-physiological signals for assessing their comprehensive mental states (Gambiraža et al., 2025). Continuous and comprehensive real-time monitoring of complex neuro-psycho-physiological parameters using a wide range of wearable sensors and ML/DL models enable accurate identification of a human’s emotional, cognitive, and behavioral states (Kesedžić et al., 2021). Electrodermal activity (EDA) or skin conductance response (SCR) tracks skin conductance associated with sympathetic activity (Posada-Quintero and Chon, 2020). Electrocardiogram (ECG) records heartbeats, while electroencephalogram (EEG) and electromyography (EMG) detect brain, muscle, and nerve activity. Photoplethysmography (PPG) estimates heart rate and blood oxygen levels (Dzedzickis et al., 2020). Respiratory belts (RB) track breathing, and electrooculogram (EOG) monitors eye movements. Accelerometers, gyroscopes, and magnetometers capture body movement. Modern technology provides up to 1,200 digital sensors to analyze 8,000 physiological and behavioral features, enabling advanced scientific machine learning applications in health and behavior analysis (Dang et al., 2023; Sabry et al., 2022). Longitudinal tracking and monitoring of human multimodal neuro-psycho-physiological features are a valuable source of information for prediction of human mental and cognitive readiness, and functional performance during unpredictable startle operations (Ćosić et al., 2019).

Stress resilience training based on stress inoculation or cognitive-behavioral skills (Palermo, 2019) applied on military and political leaders may enable enhancement of modern defense capabilities related to strategic thinking, emotional coherence, and adaptive decision-making. By investing in such a comprehensive type of training, modern political and military leaders can much easier shape the emerging global strategic threats. Without such capabilities and skills, modern civil and military leaders may exhibit short-term reactive decision patterns, reduced strategic foresight, and heightened emotional reactivity, what our adversaries can exploit using sophisticated tools and means of cognitive warfare which they may have on disposal. A stress resilience training program is highly relevant for maintaining decision-making quality, emotional stability, and ethical clarity even under sustained psychological siege, e.g., war fatigue, hybrid attacks, and political destabilization attempts. It can build mental armor of modern political, defense, and security leadership, by reinforcing psychological strength, cognitive clarity, emotional coherence, and the ability to perform successfully under high uncertainty, time pressure, and emotional threat. Enhancement of cognitive endurance and maintaining strategic depth over months/years of intense operational time can increase long-term endurance and reduce cognitive erosion due to fatigue, burnout, cognitive overload, and moral fatigue. Key techniques related to cognitive reframing, i.e., turning perceived threats into positive strategic challenges using emotionally based strategic communications (EBSC; Ćosić et al., 2026), relevant narratives, and storytelling to prevent psychological strategic collapse under pressure when cognitive and emotional reserves are exhausted. Resilient military personnel are reporting less negative and more positive affect, as well as less stress in stressful events (Niederhauser and Annen, 2023), which further emphasize the importance of the stress resilience training. Furthermore, conflicts may increasingly involve forms of psychological exhaustion, therefore without appropriate stress resilience training, modern defense will remain fragile under long-term military and political pressure. Building cognitive endurance and emotional resilience under sustained and prolonged strategic pressure will be highly relevant for protecting cognitive sovereignty and strategic readiness. Such an approach can reduce the incidence of strategic failures caused by fatigue, emotional collapse, leadership burnout and can improve the higher quality and sustainability of strategic communications and decision processes during times of a crisis, and improve public trust in modern leadership based on its ability to anticipate and withstand cognitive and psychological attacks. Training sessions focused on critical thinking skills, and the incorporation of exercises that promote analytical and critical thinking, and skills of individuals and teams can provide better decision-making processes leading to more robust performance. Utilizing cognitive neuroscience principles in training programs designed to enhance soldiers’ cognitive abilities, emotional resilience, and teamwork through understanding how brain functions can help in coping with unpredictable military threats. This proposal is consistent with broader literature on stress exposure training, mental readiness training, and emotion regulation, which frame resilience as a trainable set of cognitive and affective skills rather than a fixed trait (Driskell et al., 2018; Thompson and McCreary, 2006; Braunstein et al., 2017).

Understanding the neural mechanisms behind threat perception, attention, and situational awareness can enhance and accelerate training curve time (Ćosić et al., 2012a). Applying cognitive neuroscience can help in understanding group dynamics, leadership, and communication, and can enhance team performance and cohesion within units. On the other side, AI can be used to create advanced adaptive training to the cognitive and emotional states and overall mental readiness of soldiers, providing personalized training experiences that optimize learning and adaptation to individual learning styles and stress responses. Complex modeling and simulations can create realistic tactical scenarios for training purposes, and counteract manipulation tactics used by adversaries, allowing military personnel to practice decision-making in high-stress environments. Real-time tracking and monitoring of soldier’s physiological functions can help individuals to learn and control certain cognitive functions and processes during uncertain and unpredictable stressful situations, exploring how environmental conditions and social interactions and relationships can positively influence cognitive performance and enhance cognitive functions. Designing advanced defense training tools and means can adapt to human cognitive state in real-time by integration of virtual and augmented reality in cognitive training. Therefore, a strategic stress resilience training program may be considered an important component of modern defense and security management, providing training programs and simulation environments that consider human factors, aiming to improve training effectiveness by ensuring that simulations reflect real-world conditions and human behaviors. Revolutionizing team and individual training and operational effectiveness based on real-time simulations can enhance situational awareness and emotional regulation through a shift from individual traits to system-level cognition, while distributed decision-making should be scientifically supported by AI-based virtual assistant and adaptive human machine interfaces.

### 4.2 Mental readiness

Mental readiness as complex brain–body interactions can be assessed by comprehensive multimodal neuro-physiological measurements caused by mismatch between expected and achieved task performance. High mental workload, physical or mental stress, distracted or diverted attention, fatigue, excessive workload, decline of performance, loss of confidence, or situational disorientation can significantly impair human cognitive functions and decision-making abilities, and can cause human performance degradation (Ćosić et al., 2025). Computational assessment of mental readiness during unpredictable and unexpected stressful operations requires real-time synchronization of data related to human neuro-psycho-physiological features and corresponding environmental conditions and specific human task performance (Ćosić et al., 2022). This synchronicity is particularly important for accurate estimation of current mental readiness during military operations. Real-time cross-correlation analysis of multimodal physiological, emotional, and cognitive fluctuations with ongoing operational context and task complexity is necessary for reliable interpretations of human’s functional states and mental readiness. For example, in military aviation, a pilot’s semantic interpretation and correlation analysis of all relevant flight parameters, related to given task and mission, environmental factors such as flight weather conditions, operational and tactical situations, situational awareness, and the pilot’s neuro-psycho-physiological reactions and perturbations within a given stressful context, are highly relevant for evaluation of their flight performance. Momentarily increases in heart rate during steady state stationary flight could be used to signal potential startle situations, i.e., unexpected emotional stress, while normal heart rate increase is usually associated with take-off and landing. Therefore, context analysis is relevant for correct interpretation of different physiological fluctuations and appropriate thresholds or limits for each neuro-physiological state variable during some specific operational conditions. For example, a heart rate increase over a pre-defined upper limit could be used to signal difficulties that may require specific alarm or commander’s interventions. Understanding interactions of all these neuro-psycho-physiological multimodal features within specific operational environments is crucial for developing strategies to mitigate the negative impact of startle situations, especially for combat pilots who are operating in highly stressful, unpredictable and unexpected flight conditions. Therefore, synchronized consideration of tactical context, humans’ mental states, and their performance in real-time are a prerequisite for the reliable estimation of their mental readiness. Real-time neurophysiological measurements and their contextual interpretation may detect drift toward safety boundaries and risky performance or operators’ functional states, which may lead to potential disaster.

Machine learning (ML) and deep learning (DL) methods can significantly enhance the prediction of human mental readiness and performance by analyzing complex patterns of large neuro-psycho-physiological datasets, operational context and human performance during startle operations (Chen et al., 2022; Garriga et al., 2022; Le Glaz et al., 2021; Sabry et al., 2022). These predictive models can be continuously retrained and updated with new multimodal neurophysiological data in a real-time during specific task execution, improving accuracy and adaptability in dynamic environments. In high-stakes settings, however, the usefulness of such systems depends not only on predictive performance but also on trust calibration, and effective teaming and oversight (Reyes et al., 2025; Gonzalez et al., 2026; Kargarnovin et al., 2026). Domain knowledge related to a specific task, job, or mission is essential for the model’s accuracy and obtained models can predict individual mental readiness and performance by evaluating relationships between contextual conditions, human reactions, and mental states, helping to prevent operational errors or failures. Inclusion of irrelevant features can reduce model accuracy, emphasizing the importance of selection domain relevant features. Individual differences among military personnel who operate in extreme environments where stress, fatigue, and fear may have also substantial impact on model performance. Understanding neurobiology of stress enables cognitive adaptability, including stress resilience and stress coping strategies, providing foundations for mental readiness training to maintain self-control and operational effectiveness. Those with effective stress resilience and coping mechanisms may maintain better performance under pressure, while insights from cognitive neuroscience with AI allow better predictive models of human behavior. Such research can help in understanding the limits and capabilities of human performance under stress, which is crucial for soldier selection and mission planning. Extensive evidence suggests that stress can have a significant impact on cognitive performance and overall mental readiness, particularly in a high-stressful combat military environment. Cognitive neuroscience can also provide insights into the mechanisms underlying PTSD (Palermo, 2020b, 2021a), leading to more effective therapies and interventions for affected personnel. While combat stress has strong influence on human cognition, i.e., human decision-making process, AI-based systems are not directly affected by emotional stress in the same way as human cognition. It is particularly important advantage of potential virtual assistant based on explainable AI in highly stressful and complex combat operations. Understanding the neuroscientific background of stress on cognitive performance is crucial in high-stakes environments, such as military operations, emergency response, and high-level decision-making roles. Strategies to manage stress can help to mitigate its negative impacts and enhance overall cognitive functioning, particularly in military operations, for example through AI-based virtual assistant or adaptive human machine interfaces.

Prediction of human behavior in uncertain and unpredictable environments through machine learning analysis of large-scale datasets may support the identification of risky behaviors and decision-making vulnerabilities. Insights from cognitive neuroscience can provide the psychological aspects of warfare and conflict resolution, leading to more effective strategies that consider the unpredictable influence of volatile human factors on the outcome of military operations. Additionally, combining insights from cognitive neuroscience and AI allows developing better predictive models of human behavior and understanding how combat stress affects strategic decision-making processes in complex warfare operations. It means that AI and cognitive neuroscience can aid in strategic planning and programming of modern defense by modeling potential outcomes of different military doctrines and strategies including the psychological influence of human cognitive biases and decision-making limitations on the outcomes of complex military operations. Prevention of chronic stress may represent an important operational priority in a military affairs through regulation of cognitive overload by AI tools and means, contributing to a lower stress levels, and reduced exhaustion while maintaining high performance.

## 5 Enhancement of modern defense capabilities

Modern warfare operations are entering an age when traditional approach to kinetic power, due to the complexity and uncertainty of military operations, may benefit from deeper and more systematic understanding of human factors. In this context, redesign of modern defense and security strategy by converging neuroscience and AI, may offer an opportunity to build a more anticipatory, adaptive, and resilient security architecture. Kinetic power related to traditional combat weapons: heavy artillery, infantry fighting vehicles, main battle tanks, precise and smart ammunitions may benefit from AI-supported augmentation. Neglecting important role of the human factor in military operations may create operational and strategic vulnerabilities, since military operations are characterized by a high level of stress, uncertainty and unpredictability, lack of accurate and reliable information, emotional pressure, cognitive overload, and fatigue. Therefore, human factors concerning soldiers’ mental strength, cognitive capacities, situational awareness, attention, perception, and emotional power are highly relevant, and traditional defense paradigms may need to evolve to address not only kinetic threats but also psychological and cognitive challenges. Recent developments in cognitive neuroscience based on deeper understanding of attention, memory, stress, and emotional regulation offer better insights into human behavior and decision-making processes within stressful environments under severe emotional and cognitive pressure (Palermo, 2021b). Transition and transformation from defense kinetic power toward its cognitive enhancement is an important and complex issue. As modern warfare expands from conventional kinetic paradigms toward a new era of cognitive and hybrid threats, the convergence of cognitive neuroscience and AI emerges as a potential contributor to strategic innovation in modern defense and security affairs. This integration could be a highly attractive research topic in the unpredictable defense and security environment, with incomplete situational awareness, high mental and cognitive overload, bias in security perception, decision-making under heavy stress, and fatigue, which makes multidisciplinary scientific efforts and resources essential. The convergence and integration of advanced analytical tools and means of ML, DL, and Large Language Models (LLM) with state-of-the-art research in cognitive neuroscience, may support the analysis of complex defense and security challenges. Available tools and means of AI may enable predictive modeling, real-time monitoring and tracking of situations on the battlefield, bias detection and de-biasing algorithms, real-time disinformation detection, identification of manipulative narratives, development of emotionally aligned strategic communications, and public messaging. Accordingly Future defense investments may increasingly consider human factors, human preparedness and readiness, trustworthiness, mental agility, stress resilience, decision integrity, situational awareness, stress recovery speed, etc. Integration of cognitive strategic foresight, cognitive endurance, sustained high attention, judgment in prolonged crises, understanding of publics’ mental health, metacognition skills, awareness of cognitive limits and biases using AI tools may become increasingly relevant. Proposed transformation may increasingly consider not only what can be destroyed by kinetic power, but also how wisely, ethically, and resiliently strategic decisions can be made to avoid military conflict while simultaneously protecting strategic interests.

For illustration, cognitive-enhanced strategic deterrence can be defined as linear combination of strategic features A, B, C, and corresponding weighting factors α, β, and γ. Defense and security features A are related to kinetic power, like air defense and ground forces, B is related to cognitive features, like quality of strategic reasoning and psychological agility, leadership endurance, public resilience, and diplomatic alignment, while C is related to AI features, like predictive analytics, public sentiment analysis, disinformation detection, etc. Weighting factor α determines the weight or importance of kinetic power A, β determines the weight of the comprehensive cognitive component B, and γ denotes the weight of AI factors C. Thus, depending on the crisis scenario or strategic posture from full-scale military escalation, to rapid cyber-kinetic escalation or hybrid and disinformation warfare, toward strategic peace negotiation or public trust crisis, we can select and adapt the appropriate weighting factor combination α, β, and γ. While kinetic and deterrence capabilities remain vital, modern defense may be enhanced through the convergence of cognitive neuroscience and AI, providing prediction of potential threats and their evolution, what may contribute to anticipatory approaches in defense and security planning.

### 5.1 Strategic importance of cognitive readiness

Cognitive readiness can be achieved by a set of strategic cognitive features and their cross-functional integration related to cognitive quality distributed across different national and international institutions. Without mental resilience and readiness potential of civil and military leadership is limited, what can compromise comprehensive, timely, ethical, and resilient strategic decision-making. Furthermore, modern leadership may increasingly strengthen a set of individual cognitive strategies as well as joint cognitive governance in light of cognitive and psychological threats, requiring multi-level cognitive competence.

These cognitive capacities are measurable, trainable, and strategically relevant for modern political leadership navigating the global defense and security landscape. They are related to: (a) Cognitive flexibility, which is associated with the human ability to shift thinking in response to changing goals, contexts, or perspectives, enabling fast adaptation to geopolitical shifts, new threats, and innovation. Otherwise, due to policy rigidity and poor anticipatory capacity, operational vulnerabilities may emerge. (b) Strategic foresight, which is connected to human capacity to mentally simulate long-term consequences and interdependencies of current decisions. It is essential for proactive policy, deterrence posture, and resilience building and may be enhanced by appropriate mental readiness training and AI-based comprehensive scenario planning. (c) Cognitive empathy, as the ability to understand how others, like allies, adversaries, and public, think and feel, is crucial for diplomacy, narrative design, and disinformation resilience enabling emotional resonant and effective leadership communication. (d) Cognitive endurance, as sustained capacity for judgment and deep focus over long decision cycles, which is critical in prolonged conflicts and institutional fatigue contexts to prevent mental burnout, reactive policy and crisis overreaction. (e) Cognitive risk adjustment, as ability to balance uncertainty, probability, and strategic consequences required for de-escalation management.

Therefore, strategic power in the 21st century may not depend solely on firepower, but from multi-level cognitive readiness, emotional coherence and resilience, particularly from enhanced mental flexibility under uncertainty, strategic pressure, as well as metacognitive awareness and technological integration. Enhancing these strategically important multi-level cognitive features may represent an important priority of modern leadership, especially due to stress, speed, and ambiguity of today’s global crises.

### 5.2 Limitations, risks, and ethical considerations

While the convergence of cognitive neuroscience and artificial intelligence offers significant potential for enhancing defense and security capabilities, several important limitations, uncertainties, and ethical challenges must be explicitly acknowledged. Despite advances in neuroscience and wearable sensing technologies, the interpretation of multimodal neuro-psycho-physiological signals remains inherently probabilistic and context-dependent. Consequently, inferring constructs such as “mental readiness” or “cognitive overload” involves uncertainty, potential measurement noise, and susceptibility to false positives and false negatives. Contextual misinterpretation of physiological signals may lead to inappropriate operational or strategic decisions. AI systems, including machine learning and deep learning models, are highly dependent on the quality, representativeness, and contextual relevance of training data. In complex and dynamic operational environments, these systems may face challenges related to generalization, robustness, and adaptability. Predictive models of human behavior or cognitive states may degrade when exposed to novel or adversarial conditions. Moreover, model outputs often reflect probabilistic estimations rather than deterministic truths, requiring careful human interpretation and oversight. Continuous monitoring of neuro-physiological and behavioral data raises significant ethical and legal concerns related to privacy, autonomy, and informed consent. The collection and processing of sensitive cognitive and emotional data may create risks of misuse, unauthorized access, or coercive applications. Safeguarding individual rights while leveraging such technologies requires robust governance frameworks, strict data protection standards, and clear ethical guidelines. Technologies designed to enhance cognitive performance and resilience may also be used for manipulation, coercion, or exploitation of cognitive vulnerabilities. In the context of cognitive warfare, AI-driven tools for sentiment analysis, behavioral prediction, or targeted communication may be repurposed for disinformation, psychological operations, or influence campaigns. The integration of cognitive neuroscience and AI in defense contexts remains an emerging interdisciplinary field. While some components are supported by robust empirical evidence, others remain experimental or conceptual. Therefore, the framework proposed in this paper should be understood as a structured and evolving model that requires further empirical validation, interdisciplinary collaboration, and iterative refinement. Taken together, these limitations highlight the necessity of a cautious, transparent, and ethically grounded approach to the integration of cognitive neuroscience and AI in defense and security. Rather than diminishing the value of this convergence, acknowledging these challenges strengthens its scientific credibility and supports responsible development and application.

## 6 Conclusion

Enhancement of modern defense capabilities by convergence of cognitive neuroscience and artificial intelligence, and their integration into future command and control systems (C2I, C4ISR) may represent a significant development for future defense and security affairs. Such advanced command and control systems with augmentation of forward-thinking and decision-making capabilities may have potential to act quickly and decisively to address complex threats. The convergence of neuroscience and AI thus not only deepens our understanding of the human factor in military affairs, but also establishes the scientific architecture for next-generation operational readiness and operational superiority (Sekel et al., 2023). Policymakers can benefit from understanding neuroscientific background of crucial cognitive processes, allowing more knowledgeable and effective defense affairs that consider human factor as crucially important asset, together with technological capabilities. Joint integration of cognitive neuroscience and AI may support improved predictive modeling of human behavior, and strategic decisions under the various stressful emotional states, leading to more effective strategies to conflict prevention and resolution, particularly in situations where stress severely affects decision-making processes. Taken together, such approach outlines the importance of integration of cognitive neuroscience and AI, as well as Large Language Models (LLM), into a modern and innovative strategic defense and security affairs, potentially supporting societal resilience, adaptability, and decision superiority in future operational defense environments. Capability developments and protection of human cognition on individual and institutional level in the time of cognitive warfare by state of the art in cognitive neuroscience and AI, presented in this article, may be interpreted as a relevant step forward toward digital transformation of modern defense and security affairs.

Finally, new LLMs cognitive capabilities concerning their large-scale knowledge representations, improved pattern recognition and enhanced capacity for handling complex data structures, may support human cognitive performance, especially during the time of crises, high mental workload and high stress when human cognition regularly decline and downturn (Niu et al., 2024). Therefore, cognitive enhancing and generalization of dedicated LLMs capabilities and development of context-dependent LLM based virtual assistant and its fine-tuning regarding transformation of modern defense, and security capabilities deserves special attention, since such innovation may complement certain limitations of human cognitive processing in complex decision environments (Ćosić et al., 2026). While acknowledging various advantages of LLMs (Choi and Ferrara, 2024; Zhao et al., 2024), including their support for information extraction and synthesis, facts checking, multilingual analysis, and decision-support assistants, recent work (Griot et al., 2025) has also highlighted their important limitations in uncertainty handling, self-assessment, and metacognitive reliability in high-stakes settings. The proposed conceptual study explicitly recognizes the human brain as a strategic asset and vulnerability in modern warfare, requiring its systematic protection, monitoring, and enhancement through the convergence of neuroscience and artificial intelligence. The integration of cognitive neuroscience, AI and particularly LLM in the context of cognitive warfare and defense challenges, offers a forward-looking perspective on the transformation of defense and security policy. This does not mean that AI would replace human responsibility, but rather enhance them, which would lead to better decision-making effectiveness and improved stress management, which may contribute to enhancing human effectiveness in military and security domains.

## Data Availability

The original contributions presented in the study are included in the article/supplementary material, further inquiries can be directed to the corresponding author.

## References

[ref1] Albertella L. Kirkham R. Adler A. B. Crampton J. Drummond S. P. A. Fogarty G. J. . (2023). Building a transdisciplinary expert consensus on the cognitive drivers of performance under pressure: an international multi-panel Delphi study. Front. Psychol. 13:1017675. doi: 10.3389/fpsyg.2022.1017675, 36755983 PMC9901503

[ref2] Alharasees O. Kale U. (2025). Human factors and AI in UAV systems: enhancing operational efficiency through AHP and real-time physiological monitoring. J. Intell. Robot. Syst. 111:5. doi: 10.1007/s10846-024-02188-y

[ref3] Anderson A. J. Kiela D. Binder J. R. Fernandino L. Humphries C. J. Conant L. L. . (2021). Deep artificial neural networks reveal a distributed cortical network encoding propositional sentence-level meaning. J. Neurosci. 41, 4100–4119. doi: 10.1523/JNEUROSCI.1152-20.2021, 33753548 PMC8176751

[ref4] Baddeley A. (2012). Working memory: theories, models, and controversies. Annu. Rev. Psychol. 63, 1–29. doi: 10.1146/annurev-psych-120710-100422, 21961947

[ref5] Bebber R. J. (2025). Information Inoculation: Preparing US Warfighters for Cognitive War. Washington, D.C.: Hudson Institute.

[ref6] Botvinick M. Ritter S. Wang J. X. Kurth-Nelson Z. Blundell C. Hassabis D. (2019). Reinforcement learning, fast and slow. Trends Cogn. Sci. 23, 408–422. doi: 10.1016/j.tics.2019.02.006 31003893

[ref7] Braunstein L. M. Gross J. J. Ochsner K. N. (2017). Explicit and implicit emotion regulation: a multi-level framework. Soc. Cogn. Affect. Neurosci. 12, 1545–1557. doi: 10.1093/scan/nsx096, 28981910 PMC5647798

[ref8] Brown V. M. Morey R. A. (2012). Neural systems for cognitive and emotional processing in posttraumatic stress disorder. Front. Psychol. 3:449. doi: 10.3389/fpsyg.2012.00449, 23162499 PMC3498869

[ref9] Buzsáki G. (2006). Rhythms of the Brain. New York: Oxford University Press.

[ref10] Chen C. Han P. He F. (2022). Improving main analysis by borrowing information from auxiliary data. Stat. Med. 41, 567–579. doi: 10.1002/sim.9252, 34796519

[ref11] Choi E. C. Ferrara E. (2024). “Automated claim matching with large language models: empowering fact-checkers in the fight against misinformation,” in Companion Proceedings of the ACM web Conference 2024, (), 1441–1449. doi: 10.1145/3589335.3651910

[ref12] Cinelli M. Cresci S. Quattrociocchi W. Tesconi M. Zola P. (2022). Coordinated inauthentic behavior and information spreading on twitter. Decis. Support. Syst. 160:113819. doi: 10.1016/j.dss.2022.113819

[ref13] Claverie B. Du Cluzel F. (2022). ““Cognitive warfare”: the advent of the concept of “Cognitics”,” in The Field of Warfare. In: Cognitive Warfare: The Future of Cognitive Dominance, (NATO Science and Technology Organization, NATO-CSO-STO), 2-1–2-7.

[ref14] Cole A. Le Guyader H. (2020). Cognitive: a 6th Domain of Operations. Norfolk (VA, USA): Innovation Hub, NATO ACT Edition.

[ref15] Corbetta M. Shulman G. L. (2002). Control of goal-directed and stimulus-driven attention in the brain. Nat. Rev. Neurosci. 3, 201–215. doi: 10.1038/nrn755 11994752

[ref16] Ćosić K. Kopilaš V. Jovanović T. (2025). Importance of mental readiness in highly stressful professions. Psychiatr. Danub. 37, 132–140. doi: 10.24869/psyd.2025.132, 40886335

[ref17] Ćosić K. Popović S. Fabek I. Kovač B. Radoš M. Radoš M. . (2012a). fMRI neural activation patterns induced by professional military training. Translat. Neurosci. 3, 46–50. doi: 10.2478/s13380-012-0012-2

[ref18] Ćosić K. Popović S. Kukolja D. Horvat M. Dropuljić B. (2010). Physiology-driven adaptive virtual reality stimulation for prevention and treatment of stress related disorders. Cyberpsychol. Behav. Soc. Netw. 13, 73–78. doi: 10.1089/cyber.2009.0260, 20528296

[ref19] Ćosić K. Popović S. Šarlija M. Kesedžić I. Gambiraža M. (2022). “An approach to prediction of mental resilience in fighter pilot selection,” in Proceedings of the 1st International Conference on Cognitive Aircraft Systems (ICCAS 2022), (), 83–87.

[ref20] Ćosić K. Popović S. Šarlija M. Mijić I. Kokot M. Kesedžić I. . (2019). New tools and methods in selection of air traffic controllers based on multimodal psychophysiological measurements. IEEE Access 7, 174873–174888. doi: 10.1109/ACCESS.2019.2957357

[ref21] Ćosić K. Popović S. Wiederhold B. (2026). Emotionally based strategic communications as a new tool in defensive cognitive warfare. Front. Psychol. 17:1751406. doi: 10.3389/fpsyg.2026.1751406, 41725666 PMC12920201

[ref22] Ćosić K. Srbljinović A. Popović S. Kostović I. Judaš M. Vukšić M. (2012b). Extreme political attitudes and emotionally based strategic communications (EBSC). Journal of US-China Public Administration 9, 637–653.

[ref23] Dang T. Spathis D. Ghosh A. Mascolo C. (2023). Human-centred artificial intelligence for mobile health sensing: challenges and opportunities. R. Soc. Open Sci. 10:230806. doi: 10.1098/rsos.230806, 38026044 PMC10646451

[ref24] Doya K. (2008). Modulators of decision making. Nat. Neurosci. 11, 410–416. doi: 10.1038/nn2077, 18368048

[ref25] Draganski B. May A. (2008). Training-induced structural changes in the adult human brain. Behav. Brain Res. 192, 137–142. doi: 10.1016/j.bbr.2008.02.015, 18378330

[ref26] Driskell J. E. Salas E. Johnston J. H. Wollert T. N. (2018). “Stress exposure training: an event-based approach,” in Performance Under Stress, (London: CRC Press), 287–302.

[ref27] Dzedzickis A. Kaklauskas A. Bucinskas V. (2020). Human emotion recognition: review of sensors and methods. Sensors 20:592. doi: 10.3390/s20030592, 31973140 PMC7037130

[ref28] Ecker U. K. Lewandowsky S. Cook J. Schmid P. Fazio L. K. Brashier N. . (2022). The psychological drivers of misinformation belief and its resistance to correction. Nat. Rev. Psychol. 1, 13–29. doi: 10.1038/s44159-021-00006-y

[ref29] Eichenbaum H. (2017). Prefrontal-hippocampal interactions in episodic memory. Nat. Rev. Neurosci. 18, 547–558. doi: 10.1038/nrn.2017.74, 28655882

[ref30] Endsley M. R. (1995). Toward a theory of situation awareness in dynamic systems. Hum. Factors 37, 32–64. doi: 10.1518/001872095779049543

[ref31] Fletcher J. D. Wind A. P. (2013). “The evolving definition of cognitive readiness for military operations,” in Teaching and Measuring Cognitive Readiness, eds. O’Neil H. F. Perez R. S. Baker E. L. (Boston, MA: Springer US), 25–52.

[ref32] Gambiraža M. Popović S. Kurtak M. Jovanović T. Norrholm S. Ćosić K. (2025). Prediction of pilot performance during startle events based on neuropsychophysiological features of stress resilience and cognitive task scores. IEEE Access 13, 137525–137539. doi: 10.1109/ACCESS.2025.3594493 PMC13395618 42500601

[ref9001] Garriga R. Mas J. Abraha S. Nolan J. Harrison O. Tadros G. et al. (2022). Machine learning model to predict mental health crises from electronic health records. Nature medicine, 28, 1240–1248. doi: 10.1038/s41591-022-01811-5 PMC9205775 35577964

[ref33] Gonzalez C. Donahue K. Goldstein D. G. Heidari H. Jalali M. S. Schelble B. . (2026). Toward a science of human–AI teaming for decision making: a complementarity framework. PNAS Nexus 5:pgag030. doi: 10.1093/pnasnexus/pgag030, 41834945 PMC12983458

[ref34] Grier R. A. (2012). Military cognitive readiness at the operational and strategic levels: a theoretical model for measurement development. J. Cogn. Eng. Decis. Mak. 6, 358–392. doi: 10.1177/1555343412444606

[ref35] Griot M. Hemptinne C. Vanderdonckt J. Yuksel D. (2025). Large language models lack essential metacognition for reliable medical reasoning. Nat. Commun. 16:642. doi: 10.1038/s41467-024-55628-6, 39809759 PMC11733150

[ref37] Hart S. G. Staveland L. E. (1988). Development of NASA-TLX (task load index): results of empirical and theoretical research. Advances in Psychology 52, 139–183. doi: 10.1016/S0166-4115(08)62386-9

[ref38] Institute of Medicine (U.S.) Committee on a National Neural Circuitry Database (1991) in Mapping the Brain and its Functions: Integrating Enabling Technologies into Neuroscience Research, eds. Pechura C. M. Martin J. B. (Washington, DC: National Academies Press). 25121208

[ref39] Kargarnovin S. Hernandez C. I. Reiners D. Cruz-Neira C. Bochenek G. Karwowski W. (2026). From testbeds to high-stakes work: a review of human-AI teaming domains and teaming factors. Front. Robot. AI 13:1733942. doi: 10.3389/frobt.2026.1733942 42183027 PMC13189778

[ref40] Kesedžić I. Šarlija M. Božek J. Popović S. Ćosić K. (2021). Classification of cognitive load based on neurophysiological features from functional near-infrared spectroscopy and electrocardiography signals on n-back task. IEEE Sensors J. 21, 14131–14140. doi: 10.1109/JSEN.2020.3038032

[ref41] Kolb B. Whishaw I. Q. (2015). Fundamentals of Human Neuropsychology. New York: Worth Publishers.

[ref42] Le Glaz A. Haralambous Y. Kim-Dufor D. H. Lenca P. Billot R. Ryan T. C. . (2021). Machine learning and natural language processing in mental health: systematic review. J. Med. Internet Res. 23:e15708. doi: 10.2196/15708, 33944788 PMC8132982

[ref43] Lerner J. S. Li Y. Valdesolo P. Kassam K. S. (2015). Emotion and decision making. Annu. Rev. Psychol. 66, 799–823. doi: 10.1146/annurev-psych-010213-115043, 25251484

[ref44] Lewandowsky S. Van Der Linden S. (2021). Countering misinformation and fake news through inoculation and prebunking. Eur. Rev. Soc. Psychol. 32, 348–384. doi: 10.1080/10463283.2021.1876983

[ref45] Li Q. Molloy O. El-Fiqi H. Eves G. (2025). Applications of machine learning in assessing cognitive load of uncrewed aerial system operators and in enhancing training: a systematic review. Drones 9:760. doi: 10.3390/drones9110760

[ref46] Lieberman H. R. Bathalon G. P. Falco C. M. Morgan C. A. 3rd Niro P. J. Tharion W. J. (2005). The fog of war: decrements in cognitive performance and mood associated with combat-like stress. Aviat. Space Environ. Med. 76, C7–C14, 16018323

[ref47] Longo L. Wickens C. D. Hancock G. Hancock P. A. (2022). Human mental workload: a survey and a novel inclusive definition. Front. Psychol. 13:883321. doi: 10.3389/fpsyg.2022.883321, 35719509 PMC9201728

[ref48] Marigliano R. Ng L. H. X. Carley K. M. (2024). Analyzing digital propaganda and conflict rhetoric: a study on Russia’s bot-driven campaigns and counter-narratives during the Ukraine crisis. Soc. Netw. Anal. Min. 14:170. doi: 10.1007/s13278-024-01322-w

[ref49] Matthews G. Panganiban A. R. Wells A. Wohleber R. W. Reinerman-Jones L. E. (2019). Metacognition, hardiness, and grit as resilience factors in unmanned aerial systems (UAS) operations: a simulation study. Front. Psychol. 10:640. doi: 10.3389/fpsyg.2019.00640, 30971983 PMC6443855

[ref50] McCreight R. (2024). The war inside your mind: unprotected brain battlefields and neuro-vulnerability. Acad. Biol. 2, 1–9. doi: 10.20935/AcadBiol6156

[ref51] McEwen B. S. (2007). Physiology and neurobiology of stress and adaptation: central role of the brain. Physiol. Rev. 87, 873–904. doi: 10.1152/physrev.00041.2006, 17615391

[ref52] Menon V. (2015). “Large-scale functional brain organization,” in Brain Mapping, vol. 2 (), 449–459. doi: 10.1016/B978-0-12-397025-1.00024-5

[ref53] Miller B. L. Cummings J. L. (2018). The Human Frontal Lobes: Functions and Disorders. 3rd Edn New York: The Guilford Press.

[ref54] Moreno J. D. (2006). Mind Wars: Brain Research and National Defense. New York, NY: Dana Press.

[ref9002] Morese R. Stanziano M. Palermo S. (2018). Commentary: Metacognition and Perspective-Taking in Alzheimer’s Disease: A Mini-Review. Front Psychol. 9:2010. doi: 10.3389/fpsyg.2018.02010 Erratum in: Front Psychol. 11:2157. doi: 10.3389/fpsyg.2020.02157 30405494 PMC6204392

[ref55] National Research Council (2015). Measuring Human Capabilities: An Agenda for Basic Research on the Assessment of Individual and Group Performance Potential for Military Accession. Washington, DC: The National Academies Press.

[ref56] Niederhauser M. Annen H. (2023). “Resilience and resilience training: focus on military science,” in the Positive Psychology in the Military, ed. Annen H. (Lausanne: Peter Lang International Academic Publishers), 27–54.

[ref57] Niu Q. Liu J. Bi Z. Feng P. Peng B. Chen K. . (2024). Large language models and cognitive science: a comprehensive review of similarities, differences, and challenges. arXiv preprint arXiv:2409.02387.

[ref58] Pacheco D. Hui P. M. Torres-Lugo C. Truong B. T. Flammini A. Menczer F. (2021). “Uncovering coordinated networks on social media: methods and case studies,” in Proceedings of the International AAAI Conference on Web and Social Media, vol. 15 (), 455–466.

[ref59] Palermo S. (2018). Crossing in the red zone: mTBI/concussion and PTSD in the context of war. EC Psychol. Psychiatr. 7, 287–288.

[ref60] Palermo S. (2019). Healing from war trauma and moving on: creative approaches and psychological therapy based on the neurobiology of PTSD. EC Psychol. Psychiatr. 2, 10–11.

[ref61] Palermo S. (2020a). COVID-19, paura della morte in uno scenario VUCA: lezioni di psicologia militare. Report Difesa.

[ref62] Palermo S. (2020b). From world war I to the attack in Nassiriya: what we knew and what we learned about post-traumatic stress disorder. EC Psychol. Psychiatr. 9, 1–3.34557877

[ref63] Palermo S. (2020c). La psicologia delle folle: da Gustave Le Bon alla sociologia dei comportamenti collettivi. Report Difesa.

[ref64] Palermo S. (2021a). Desert Storm, 30 anni fa: sindrome della guerra del Golfo e alterazioni del sistema nervoso centrale. San Martino Valle Caudina (AV), Italy: Report Difesa.

[ref65] Palermo S. (2021b). Special in uniform: forze armate israeliane ed impiego di soldati con abilità speciali. Report Difesa.

[ref9004] Palermo S. Di Fazio C. Scaliti E. Stanziano M. Nigri A. Tamietto M. (2025). Cortical excitability and the aging brain: toward a biomarker of cognitive resilience. Front Psychol. 16:1542880. doi: 10.3389/fpsyg.2025.1542880 40040658 PMC11878273

[ref9005] Palermo S. Lopiano L. Morese R. Zibetti M. Romagnolo A. Stanziano M. et al. (2018). Role of the Cingulate Cortex in Dyskinesias-Reduced-Self-Awareness: An fMRI Study on Parkinson’s Disease Patients. Frontiers in psychology, 9:1765. doi: 10.3389/fpsyg.2018.01765 30294293 PMC6159748

[ref9006] Palermo S. Morese R. Zibetti M. Dematteis F. Sirgiovanni S. Stanziano M. et al. (2017). Impulse control disorder and response-inhibition alterations in Parkinson’s disease. A rare case of totally absent functionality of the medial-prefrontal cortex and review of literature. Journal of advanced research, 8, 713–716. doi: 10.1016/j.jare.2017.09.004 29034115 PMC5633757

[ref66] Pastor A. (2025). Cognitive warfare. HAL preprint, hal-04420986v2. https://hal.science/hal-04420986v2

[ref67] Pessoa L. (2015). Précis on the cognitive-emotional brain. Behav. Brain Sci. 38:e71. doi: 10.1017/S0140525X14000120, 24914882

[ref68] Pomerantsev P. (2019). This Is Not Propaganda: Adventures in the War Against Reality. London: Faber & Faber.

[ref69] Popović S. Horvat M. Kukolja D. Dropuljić B. Ćosić K. (2009). Stress inoculation training supported by physiology-driven adaptive virtual reality stimulation. Stud. Health Technol. Inform. 144, 50–54, 19592729

[ref70] Posada-Quintero H. F. Chon K. H. (2020). Innovations in electrodermal activity data collection and signal processing: a systematic review. Sensors 20:479. doi: 10.3390/s20020479, 31952141 PMC7014446

[ref71] Posner M. I Petersen S. E. (1990). The attention system of the human brain. Annu. Rev. Neurosci. 13, 25–42. doi: 10.1146/annurev.ne.13.030190.000325, 2183676

[ref72] Ramallo-Luna M. A. Gonzalez-Torre S. Rodríguez-Mora Á. de la Torre G. G. (2025). Neurocognitive factors of new drone pilots: identifying candidates with expert potential. Comput. Hum. Behav. Rep. 19:100705. doi: 10.1016/j.chbr.2025.100705, 38826717

[ref73] Reale C. Salwei M. E. Militello L. G. Weinger M. B. Burden A. Sushereba C. . (2023). Decision-making during high-risk events: a systematic literature review. J. Cogn. Eng. Decis. Mak. 17, 188–212. doi: 10.1177/15553434221147415, 37823061 PMC10564111

[ref74] Reyes J. Batmaz A. U. Kersten-Oertel M. (2025). Trusting AI: does uncertainty visualization affect decision-making? Front. Comput. Sci. 7:1464348. doi: 10.3389/fcomp.2025.1464348

[ref75] Robbins T. W. Arnsten A. F. (2009). The neuropsychopharmacology of fronto-executive function: monoaminergic modulation. Ann. Rev. Neurosci. 32, 267–287. doi: 10.1146/annurev.neuro.051508.135535, 19555290 PMC2863127

[ref76] Sabry F. Eltaras T. Labda W. Alzoubi K. Malluhi Q. (2022). Machine learning for healthcare wearable devices: the big picture. J. Healthc. Eng. 2022, 1–25. doi: 10.1155/2022/4653923, 35480146 PMC9038375

[ref77] Schubert J. Brynielsson J. Nilsson M. Svenmarck P. (2018). “Artificial intelligence for decision support in command and control systems,” in 23rd International Command and Control Research & Technology Symposium “Multi-Domain C2”, vol. 2 (), 18–33.

[ref9003] Sekel N. M. Beckner M. E. Conkright W. R. LaGoy A. D. Proessl F. Lovalekar M. . (2023). Military tactical adaptive decision making during simulated military operational stress is influenced by personality, resilience, aerobic fitness, and neurocognitive function. Frontiers in psychology, 14:1102425. doi: 10.3389/fpsyg.2023.1102425 36844343 PMC9944034

[ref78] Sharma K. Zhang Y. Ferrara E. Liu Y. (2021). “Identifying coordinated accounts on social media through hidden influence and group behaviours,” in Proceedings of the 27th ACM SIGKDD Conference on Knowledge discovery & data mining, (), 1441–1451.

[ref79] Sherman S. (2016). Thalamus plays a central role in ongoing cortical functioning. Nat. Neurosci. 19, 533–541. doi: 10.1038/nn.4269, 27021938

[ref80] Shneiderman B. (2020). Human-centered artificial intelligence: reliable, safe & trustworthy. Int. J. Hum.-Comput. Interact. 36, 495–504. doi: 10.1080/10447318.2020.1741118

[ref81] Smith C. D. Cooper A. D. Merullo D. J. Cohen B. S. Heaton K. J. Claro P. J. . (2019). Sleep restriction and cognitive load affect performance on a simulated marksmanship task. J. Sleep Res. 28:e12637. doi: 10.1111/jsr.12637, 29171171

[ref82] Taddeo M. Floridi L. (2018). How AI can be a force for good. Science 361, 751–752. doi: 10.1126/science.aat5991, 30139858

[ref83] Thompson R. R. Jones N. M. Holman E. A. Silver R. C. (2019). Media exposure to mass violence events can fuel a cycle of distress. Sci. Adv. 5:eaav3502. doi: 10.1126/sciadv.aav3502, 31001584 PMC6469939

[ref84] Thompson M. M. McCreary D. R. (2006). “Enhancing mental readiness in military personnel,” in RTO Meeting Proceedings MP-HFM-134 on Human Dimensions in Military Operations – Military Leaders' Strategies for Addressing Stress and Psychological Support, 24–26 April 2006, Brussels, Belgium, (). (Neuilly-sur-Seine: RTO/NATO), 4-1–4-12

[ref85] Trabucco L. Larsen E. S. (2025) Artificial Intelligence in Command and Control. Available online at: https://cms.polsci.ku.dk/english/publications/artificial-intelligence-in-command-and-control/

[ref86] van Diggelen J. Metcalfe J. S. van den Bosch K. Neerincx M. Kerstholt J. H. (2023). Role of emotions in responsible military AI. Ethics Inf. Technol. 25:17. doi: 10.1007/s10676-02309695-w

[ref87] Vernon D. Metta G. Sandini G. (2007). A survey of artificial cognitive systems: implications for the autonomous development of mental capabilities in computational agents. IEEE Trans. Evol. Comput. 11, 151–180. doi: 10.1109/TEVC.2006.890274

[ref88] Young M. S. Brookhuis K. A. Wickens C. D. Hancock P. A. (2015). State of science: mental workload in ergonomics. Ergonomics 58, 1–17. doi: 10.1080/00140139.2014.956151 25442818

[ref89] Zhao X. Deng Y. Yang M. Wang L. Zhang R. Cheng H. . (2024). A comprehensive survey on relation extraction: recent advances and new frontiers. ACM Comput. Surv. 56, 1–39. doi: 10.1145/3674501

